# Is there room for ethics of authenticity in psilocybin research?

**DOI:** 10.1016/j.nsa.2025.105519

**Published:** 2025-03-24

**Authors:** Stefan Jerotic

**Affiliations:** a Clinic for Psychiatry, University Clinical Centre of Serbia, Belgrade, Serbia; b Faculty of Medicine, University of Belgrade, Belgrade, Serbia

Essential questions are raised by the concept of authenticity – central to existential philosophy and virtue ethics – particularly in the context of psychedelic experiences. The key question is: do changes in personality and physiology induced by psilocybin express the core of a person's being, or do they merely represent a transient alteration imposed by an external agent? In other words, does undergoing a psychedelic experience threaten a person's authenticity by causing them to lose touch with their true self?

Recent work by Godfrey et al. (2024) on the psychological and physiological moderators of psychedelic-induced personality change among healthy volunteers has shown that even a single psilocybin dose can lead to enduring shifts in personality. Using a robust placebo-controlled design, the authors demonstrated reductions in neuroticism and an increase in agreeableness. This builds on a body of recent work suggesting that a single intake may lead to personality effects that last for years (Knudsen, 2023). However, it should be taken into account that findings on psychedelic-induced personality changes require cautious interpretation. For instance, while early influential studies from the Hopkins group reported increased openness following psilocybin administration, they did not observe reliable changes in neuroticism (MacLean et al., 2011).

Nevertheless, findings of Godfrey et al. (2024) reinforce the possibility that psychedelics – under certain psychological and physiological conditions – may reliably influence specific personality domains, such as neuroticism and agreeableness. These effects align in part with the reductions in neuroticism induced by SSRIs in healthy individuals, though SSRIs typically produce changes that are subtler and seem to unfold gradually over time (Ilieva, 2015). Importantly, SSRIs can also lead to less desirable outcomes, such as emotional blunting or impoverished affect, significantly impacting an individual's sense of self (Goodwin et al., 2017). Thus, while both psychedelics and SSRIs raise ethical questions regarding personality change, they differ crucially in the immediacy, intensity, and subjective experience of these changes.

All neurotechnologies and pharmacological agents raise questions about the kind of “self” we wish to cultivate (Jerotic, 2024). Some years ago, antidepressants were viewed not only as treatments but also as potential agents of enhancement. This shift has sparked significant debates among psychiatrists, philosophers, and patients (Elliott, 2004). However, while antidepressants appear to slowly shift cognitive and emotional baselines, giving individuals time to adapt and integrate changes, psilocybin's effects tend to be immediate and profound. This immediacy can blur the line between therapeutic intervention and enhancement, making it difficult to determine whether these changes promote self-alignment or disrupt one's pre-existing sense of identity. Psilocybin's potential to induce rapid personality changes may either align with or clash with personal, societal, and cultural understandings of what constitutes authentic personal growth (Svenaeus, 2009). Unlike interventions that promote gradual change, the swift biological and personality reconfiguration associated with psychedelics presents a unique ethical dilemma: do such changes respect the continuity of an individual's self-concept, or do they create dissonance between who they were and who they become?

The rapid personality changes that seem to be induced by psilocybin suggest a potential for both profound self-exploration and the risk of disorientation. The rapid personality changes induced by psilocybin suggest a potential for both profound self-exploration and the risk of disorientation. These outcomes may not be mutually exclusive, as Godfrey et al. (2024) demonstrate that reductions in neuroticism are moderated by psychological factors, such as the meaningfulness of the experience and the dread of ego dissolution.

It is important to note, however, that the concept of authenticity – and by extension, the idea of a “true self” – is subject to ongoing philosophical and neuroethical debate. While some theorists propose that a stable core identity or essential “true self” grounds authenticity, others argue that the self is fundamentally fluid, shaped continuously through personal experiences and interventions (Pugh et al., 2017). The ethical implications of psychedelic-induced personality change must therefore engage carefully with this complexity, acknowledging that authenticity may involve both self-discovery and active self-creation (Langlitz and Gearin, 2024).

Taking everything into account, participants undergoing psilocybin research should be aware of the potential for these interventions to reshape their broader sense of identity. Ultimately, the ethical discussion surrounding authenticity in the context of psychedelic-induced personality change cannot be separated from the broader social and psychological frameworks in which these substances are used. Neuroscientific research into the mechanisms of these changes provides valuable insights, but it must be integrated with a philosophical understanding of what it means for a change to be authentic.

## Declaration of competing interest

None.
